# Peripheral blood mitochondrial DNA content in relation to circulating metabolites and inflammatory markers: A population study

**DOI:** 10.1371/journal.pone.0181036

**Published:** 2017-07-13

**Authors:** Judita Knez, Vannina G. Marrachelli, Nicholas Cauwenberghs, Ellen Winckelmans, Zhenyu Zhang, Lutgarde Thijs, Jana Brguljan-Hitij, Michelle Plusquin, Christian Delles, Daniel Monleon, Josep Redón, Jan A. Staessen, Tim S. Nawrot, Tatiana Kuznetsova

**Affiliations:** 1 Research Unit Hypertension and Cardiovascular Epidemiology, KU Leuven Department of Cardiovascular Sciences, University of Leuven, Leuven, Belgium; 2 Department of Hypertension, Division of Internal Medicine, University Medical Centre Ljubljana, Ljubljana, Slovenia; 3 Metabolomic and Molecular Image Laboratory, Fundación Investigatión Clínico de Valencia (INCLIVA), Valencia, Spain; 4 Centre for Environmental Sciences, Hasselt University, Diepenbeek, Belgium; 5 Institute of Cardiovascular and Medical Sciences, University of Glasgow, Glasgow, Scotland, United Kingdom; 6 KU Leuven Department of Public Health, Occupational and Environmental Medicine, University of Leuven, Leuven, Belgium; Vanderbilt University Medical Center, UNITED STATES

## Abstract

Mitochondrial DNA (mtDNA) content might undergo significant changes caused by metabolic derangements, oxidative stress and inflammation that lead to development and progression of cardiovascular diseases. We, therefore, investigated in a general population the association of peripheral blood mtDNA content with circulating metabolites and inflammatory markers. We examined 310 subjects (50.6% women; mean age, 53.3 years) randomly selected from a Flemish population. Relative mtDNA content was measured by quantitative real-time PCR in peripheral blood cells. Peak circulating metabolites were quantified using nuclear magnetic resonance spectroscopy. The level of inflammation was assessed via established inflammatory markers. Using Partial Least Squares analysis, we constructed 3 latent factors from the 44 measured metabolites that explained 62.5% and 8.5% of the variance in the contributing metabolites and the mtDNA content, respectively. With adjustments applied, mtDNA content was positively associated with the first latent factor (*P* = 0.002). We identified 6 metabolites with a major impact on the construction of this latent factor including HDL3 apolipoproteins, tyrosine, fatty acid with αCH2, creatinine, β-glucose and valine. We summarized them into a single composite metabolite score. We observed a negative association between the composite metabolic score and mtDNA content (*P* = 0.001). We also found that mtDNA content was inversely associated with inflammatory markers including hs-CRP, hs-IL6, white blood cell and neutrophil counts as well as neutrophil-to-lymphocyte ratio (*P*≤0.0024). We demonstrated that in a general population relative peripheral blood mtDNA content was associated with circulating metabolites indicative of perturbed lipid metabolism and with inflammatory biomarkers.

## Introduction

Atherosclerotic vascular disease is the leading cause of morbidity and mortality [1]. Development and progression of atherosclerosis in vascular wall is associated with complex processes of metabolic derangements, oxidative stress and inflammation [2]. Several lines of evidence imply that mitochondrial DNA (mtDNA) damage and subsequent organelle dysfunction might be the link between these processes [3–5].

Each mitochondrion contains several mtDNA molecules, susceptible to oxidative damage [3]. Maintenance of sufficient mtDNA copy numbers (also referred as mtDNA content) is important for cellular homeostasis [6]. Accumulation of mtDNA damage can perturb its replication and results in decreased mtDNA content [3, 7]. Previous experimental studies showed that overproduction of reactive oxygen species (ROS) by mitochondrial enzymes causes mtDNA damage and activation of proatherogenic inflammasomes [3, 8]. In murine aortic wall, decreased mtDNA content preceded the development of detectable atherosclerotic lesions [3]. In humans, lower mtDNA content measured in peripheral blood cells was observed in patients with coronary heart disease and hyperlipidaemia as compared to healthy controls [9, 10]. Of notice, a positive correlation was reported between mtDNA content measured in atherosclerotic plaque tissue and in peripheral blood cells [9].

On the other hand, a metabolic signature indicative of mitochondrial dysfunction in the elderly subjects was associated with adverse cardiovascular outcome independently of conventional risk factors [11]. Accordingly, quantification of circulating metabolites improved prediction of subclinical atherosclerosis in young adults [12]. Thus, it is plausible that metabolic profiles and inflammation reflecting increased oxidative stress might be related to mitochondrial dysfunction. However, population data exploring associations of mtDNA content and circulating metabolites and inflammatory markers are scarce. In the present study we, therefore, investigated in a general population sample whether mtDNA content measured in peripheral blood cells is associated with circulating metabolites and markers of inflammation. In our study, we explored the relation between mtDNA content and complete metabolite profile measured by proton nuclear magnetic resonance (1H-NMR) spectroscopy rather than to perform association analyses of mtDNA with some pre-selected metabolites.

## Materials and methods

### Study participants

As described in detail previously [13, 14], the Flemish Study on Environment, Genes and Health Outcomes (FLEMENGHO) is a large ongoing family-based population study. The Ethics Committee of the University of Leuven approved the FLEMENGHO study. All participants gave written informed consent. From August 1985 until December 2002, we identified a random population sample stratified by sex and age from a geographically defined area in northern Belgium. Households, defined as people who lived at the same address, were the sampling unit. We numbered households consecutively and generated a random number list by use of SAS random function. Households with a number matching the list were invited; household members older than 18 years were eligible. The initial response rate was 78%. From 2005–2010, 316 former participants were re-examined at the field centre, in whom we also measured peripheral blood mtDNA content, circulating serum metabolites and inflammatory markers. Of those, we excluded 6 subjects from analysis because the quality of mtDNA measurement (n = 2) or metabolites measurement (n = 4) was insufficient. Thus, we included into the analysis 310 subjects in whom mtDNA content, circulating metabolites and inflammatory markers were successfully measured.

### Clinical measurements

The complete protocol of the clinical measurements was described in details elsewhere [13, 14]. Briefly, on the day of the examination, participants completed a validated questionnaire inquiring into life-style, medical history and intake of medications. Trained nurses measured anthropometric characteristics and blood pressure five times consecutively to the nearest 2 mmHg after the participants had rested for 5 minutes in the sitting position. Hypertension was a blood pressure of at least 140 mmHg systolic or 90 mmHg diastolic or use of antihypertensive drugs. Body mass index was weight in kilograms divided by the square of height in meters. Diabetes mellitus was determined by self-reported diagnosis, fasting glucose level of at least 126 mg/dL, or use of antidiabetic agents. Venous blood samples were drawn for measurement of blood glucose, serum creatinine, cholesterol as well as “classical” markers of inflammation such as high sensitivity C-reactive protein (hs-CRP) and interleukin-6 (hs-IL-6). A differential blood cell count was performed using an automated analyser.

### Metabolite analysis

To measure the level of peak circulating metabolites in the participant’s serum samples we used proton nuclear magnetic resonance (1H-NMR) spectroscopy as described previously [15]. A detailed description of the metabolite detection, identification and quantification is provided in the *S1 Appendix Methods*. Briefly, after initial preparation samples were transferred into high resolution NMR tubes. For all samples 1H-NMR signals were recorded in a Bruker Avance DRX 600 spectrometer (Rheinstetten, Germany). The obtained 1H-NMR signals were Fourier transformed into frequency spectra. Chemical shift referencing on a standard signal was performed in all spectra and used together with available spectral databases to identify the metabolites. Metabolite signals were integrated and quantified using in-house MATLAB peak-fitting routines.

### Measurement of mtDNA content

To determine the peripheral blood mtDNA content we used a real time quantitative polymerase chain reaction (qPCR) assay, as described previously [14]. A detailed description of the qPCR assay and calculation of the relative mtDNA quantity (the mtDNA content) is provided in the *S1 Appendix Methods* and *S1 Table*. Briefly, we extracted total genomic DNA from peripheral blood samples, using the QIAmp DNA Mini Kit (QIAgen, Hilden, Germany). Using qPCR, we amplified two stable mtDNA sequences (mitochondrially encoded NADH dehydrogenase 1 (MT-ND1) and mitochondrial forward primer from nucleotide 3212 and reverse primer from nucleotide 3319 (MTF3212/R3319) and one reference nuclear gene (acidic ribosomal phosphoprotein P0 (RPLP0). For consistency, all samples were run in triplicates. Cycle threshold (Ct) values of the 2 mitochondrial DNA sequences were normalized relative to the nuclear gene using qBase quantification software (Biogazelle, Zwijnaarde, BE) [16]. The qBase software uses the relative normalized values based on the delta-delta-Ct method taking multiple sequences, and the inter-run calibrators into account. The coefficient of variation between triplicate measurements within the same run was <0.5% for each of the amplified sequences, and 4.66% for the interrun samples.

### Statistical analysis

For database management and statistical analysis, we used SAS software, version 9.4 (SAS Institute, Cary, NC) and JMP Genomics, version 6.1 (SAS Institute, Cary, NC). We compared means and proportions by the t-test and the χ^2^ test, respectively. Significance was *P*<0.05 on two-sided test.

We normalized the distributions of all the metabolites by a logarithmic transformation and by a rank based inverse normal transformation [17]. We used Partial Least Squares analysis (PLS) to identify circulating metabolites associated with peripheral blood mtDNA content [18]. We chose this method due to its ability to deal with highly correlated predictors (metabolites). With PLS regression we created linear combinations (*latent factors*) of highly inter-correlated normalized predictors (metabolites) in a way that maximize the covariance between the metabolites and the outcome variable (mtDNA content). This approach differs from the classical multiple linear regression approach in that only the relevant part of the information present in all the metabolites is used to construct the different latent factors for the prediction of outcome (mtDNA content). We then identified the minimum number of latent factors that explained a substantial proportion of variation for both predictor and outcome variables and was not significantly different from the model with the minimum prediction error sum of squares (PRESS) value. The selected latent factors are used in association instead of the original individual predictors (metabolites). The importance of each metabolite in the construction of the latent factors was assessed from the variable influence on projection (VIP) scores of Wold. In the present analysis, metabolites with a VIP ≥ 1.5 were considered influential.

We summarized the influential metabolites with a VIP ≥ 1.5 into a single normally distributed composite score using principal component analysis. We used a mixed model to test the association of mtDNA content with latent factors, the composite metabolite score, logarithmically transformed selected metabolites and inflammatory markers accounting for previously identified covariates [14] and for non-independence of observations within families. We further explored correlations between pairs of selected metabolites linked to mtDNA content by Pearson correlation analysis.

## Results

### Characteristics of participants

The 310 white European participants included 157 (50.6%) women, 136 (43.9%) hypertensive and 8 (2.6%) diabetic subjects. Mean age (±SD) was 51.3±16.5 years and ranged from 17 to 84 years. Table 1 summarizes the clinical and biochemical characteristics of the participants by sex. Compared to women, men had higher diastolic blood pressure, serum creatinine and triglycerides (Table 1). Alcohol use was also more frequently reported in men (Table 1). On the other hand, women had higher heart rate, total and high density lipoprotein (HDL) serum cholesterol, hs-CRP, lymphocyte and platelet counts (Table 1).

**Table 1:** Clinical characteristics of participants by sex.

Characteristic	Clinical Measurements		
	Women (n = 157)	Men (n = 153)	*P Value*
*Anthropometrics*			
Age (years)	51.5±16.4	51.1±16.6	0.80
Body mass index (kg/m2)	26.7±5.15	27.1±4.46	0.49
Systolic pressure (mmHg)	128.4±18.4	131.1±15.8	0.17
Diastolic pressure (mmHg)	78.2±8.88	81.5±10.1	0.003
Heart rate (beats/min)	62.3±9.51	59.9±10.4	0.033
*Questionnaire data*			
Current smoking	25 (15.9)	37 (24.2)	0.070
Drinking alcohol	28 (17.8)	87 (56.9)	< .0001
Hypertensive	66 (42.0)	70 (45.8)	0.51
Treated for hypertension	51 (32.5)	47 (30.7)	0.74
Diabetes	3 (1.91)	5 (3.27)	0.45
*Biochemical data*			
Plasma glucose (mmol/l)	4.85±0.78	5.03±0.94	0.80
Serum creatinine (μmol/l)	73.7±12.8	89.5±13.1	< .0001
Serum triglycerides (mmol/l)	1.68±0.85	2.06±1.20	0.001
Serum total cholesterol (mmol/l)	5.34±0.93	5.05±0.89	0.005
LDL cholesterol (mmol/l)	3.21±0.80	3.08±0.79	0.14
HDL cholesterol (mmol/l)	1.56±0.36	1.28±0.27	< .0001
hs-CRP (mg/L)	1.95 (0.80 to 10.2)	1.24 (0.62 to 2.88)	< .0001
hs-IL6 (pg/mL)	1.55 (0.65 to 3.71)	1.44 (0.62 to 3.47)	0.41
*Blood cell count*			
Platelets (x10^9^/L)	253.6±56.7	212.7±49.1	< .0001
White blood cells (x10^9^/L)	6.53±1.74	6.34±1.70	0.33
Neutrophils (x10^9^/L)	3.80±1.32	3.72±1.19	0.58
Lymphocytes (x10^9^/L)	2.06±0.64	1.88±0.68	0.019
Neutrophil-to-lymphocyte ratio	1.97±0.79	2.16±0.89	0.055
mtDNA content^a^	1.08±0.40	1.01±0.39	0.14

Values are mean (±SD), geometric mean (10% to 90% interval), or number of subjects (%). HDL, high density lipoprotein; hs-CRP, high sensitivity C-reactive protein; hs-IL6, high sensitivity interleukin-6; LDL, low density lipoprotein; mtDNA, mitochondrial deoxyribonucleic acid.

^a^ Relative ratio of 2 mtDNA sequences (mitochondrially encoded NADH dehydrogenase 1 (MT-ND1) and mitochondrial forward primer from nucleotide 3212 and reverse primer from nucleotide 3319 (MTF3212/R3319)) to a single reference nuclear gene (acidic ribosomal phosphoprotein P0 (RPLP0)).

### PLS analyses

**Fig 1:**
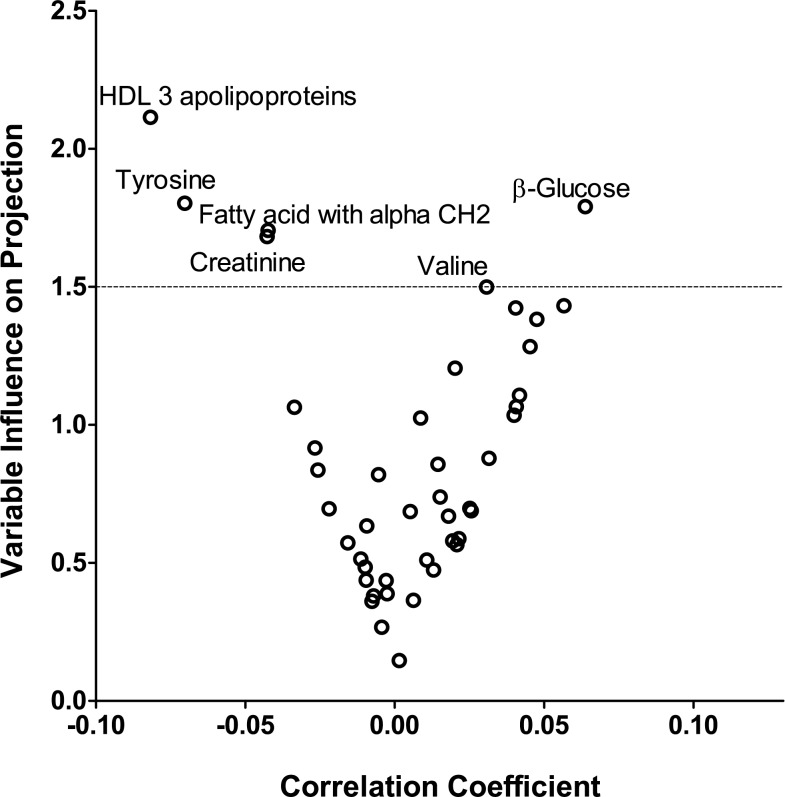
V-plot for partial least squares models generated with extracted variable influences on projection and correlation coefficient values in all participants. Spots with high variable influences on projection (≥1.5) are named at the ends of the two arms of ‘V’.

Because of the high intra-correlation of the 44 circulating metabolites (S1 Fig), we used PLS regression to compose uncorrelated latent factors derived from the measured metabolites as described in the statistical analysis section. The best-fit model includes the minimal number of latent factors that predict a significant proportion of the mtDNA content and does not differ from the model with minimal PRESS. In our analysis, we identified that such a model included three latent factors that accounted for 62.5% of the overall variance in the circulating metabolites and 8.6% of the variance in mtDNA content (*P* < 0.001 for both). The role of each of the metabolites in constructing of the latent factors is reflected by the latent factor loading and the variable influence on projection (VIP) scores. *S2 Table* lists factor loadings for each of the metabolites included in each of the latent factors. Next, we identified the most important metabolites in the construction of the latent factors using V-plot (Fig 1). This plot shows the VIP scores versus the centred and scaled correlation coefficients between blood mtDNA content and circulating metabolites. We considered metabolites with values for VIP > 1.5 to be important for the latent factor construction. Metabolites with the highest VIP values and correlating positively with mtDNA were β-glucose and valine. In Fig 1 these metabolites correspond to spots in the upper right quadrant. Metabolites with the highest VIP values and correlating negatively with mtDNA were HDL3 apolipoproteins, tyrosine, fatty acid with αCH2 and creatinine. In Fig 1 these metabolites correspond to spots in the upper left quadrant.

Table 2 shows results of univariate association of mtDNA content with each of the six metabolites that were important for construction of the latent factors. Pairwise correlations between the selected metabolites are shown in Table 3. HDL3 apolipoproteins showed strong positive correlations with tyrosine (r = 0.95; *P* < 0.0001) and creatinine (r = 0.49; *P* < 0.0001) whereas valine showed strong negative correlation with creatinine (r = –0.64; *P* < 0.0001) and fatty acid with αCH2 (r = –0.68; *P* < 0.0001; Table 3). Moreover, we noticed that there are overlapping resonances arising from HDL3 apolipoproteins and tyrosine metabolites (Table 2). Therefore, we further explored univariate associations of mtDNA content with other tyrosine spectral regions (*S3 Table*). We did not observe significant associations of mtDNA with the tyrosine metabolites from the spectral regions that did not overlap with HDL3 apolipoproteins (*P* ≥ 0.18).

**Table 2:** Associations between the mtDNA content and the selected metabolites (VIP>1.5).

Explanatory Variable	Residual Water Peak Region (ppm)	mtDNA Content			
		Parameter Estimate ± SE	95% CI	*P*	P*
HDL3 apolipoproteins	6.50–7.50	-0.41**±**0.14	-0.67 to -0.14	0.003	0.005
Tyrosine	6.88–6.90	-0.33**±**0.14	-0.61 to -0.063	0.016	0.021
β-Glucose	3.22–3.26	0.34**±**0.14	0.088 to 0.60	0.008	0.014
Fatty acid with αCH2	2.19–2.23	-0.52±0.22	-0.95 to -0.091	0.018	0.006
Creatinine	4.04–4.06	-0.71±0.24	-1.19 to -0.24	0.004	0.004
Valine	0.97–0.99	0.98±0.38	0.23 to 1.73	0.010	0.012

Explanatory variables were normalized by a logarithmic transformation. Parameter estimates, corresponding SE and 95% CI are associated with a doubling of the metabolites. *P* values are for models with explanatory variables normalized by a logarithmic transformation.

P* values are for models with explanatory variables normalized by a rank transformation.

HDL, high density lipoprotein; mtDNA, mitochondrial deoxyribonucleic acid; SE, standard error; CI, confidence interval; VIP, variable influence on projection.

**Table 3:** Correlation matrix for the selected metabolites (VIP>1.5).

Metabolites	HDL3 apolipoproteins	Tyrosine	β-Glucose	Fatty acid with αCH2	Creatinine
Tyrosine	0.95**	/			
β-Glucose	-0.054	-0.15*	/		
Fatty acid with alpha CH2	-0.14*	-0.19*	-0.35**	/	
Creatinine	0.49**	0.37**	-0.21*	0.31**	/
Valine	-0.10	-0.009	0.31**	-0.68**	-0.64**

Significance

*P<0.05

**P<0.0001.

HDL, high density lipoprotein; VIP, variable influence on projection.

### Multivariable adjusted associations between peripheral blood mtDNA content, metabolite derived latent factors, the composite metabolite score and inflammatory markers

We adjusted the models for previously identified important covariables of mtDNA content in our population such as sex, age, white blood cell and platelet counts as well as for non-independence of observations within families. After full adjustment, peripheral blood mtDNA was positively associated with the latent factor 1 (effect size: 0.070±0.022, *P* = 0.002; Table 4). We did not observe associations between the mtDNA content and two other latent factors.

**Table 4:** Multivariable-adjusted associations of mtDNA content with metabolic latent factors and inflammatory markers.

	mtDNA Content		
Parameter	Parameter Estimate±SE	95%CI	*P V*alue
*Metabolic latent factors*			
Factor 1 (+2.70)	0.070±0.022	0.027 to 0.11	0.002
Factor 2 (+3.32)	0.040±0.020	-0.002 to 0.083	0.059
Factor 3 (+2.52)	0.038±0.023	-0.009 to 0.083	0.11
*Inflammatory markers*			
hs-CRP (doubling)	-0.063±0.019	-0.10 to -0.024	0.002
hs-IL6 (doubling)	-0.072±0.024	-0.12 to -0.023	0.004
White blood cells (+1.72x10^9^/L)	-0.086±0.022	-0.12 to -0.034	0.0002
Neutrophils (+1.26x10^9^/L)	-0.088±0.025	-0.13 to -0.038	0.0001
Lymphocytes (+0.67x10^9^/L)	-0.040±0.020	-0.067 to 0.004	0.073
NLR (+0.84)	-0.050±0.021	-0.092 to -0.007	0.024

Parameter estimates and corresponding SE and 95%CI are expressed for a 1 SD increase in the explanatory variables. For hs-CRP and hs-IL-6 parameter estimates and corresponding SE and 95%CI are associated with a doubling of the inflammatory marker. Models for latent factors were adjusted for age, sex, white blood cell count, platelet count and family clusters. Models for inflammatory markers were adjusted for age, sex, platelet count, and family clusters. mtDNA, mitochondrial deoxyribonucleic acid; NLR, neutrophil-to-lymphocyte ratio; SE, standard error; CI, confidence interval; hs-CRP, high sensitivity C-reactive protein; hs-IL6, high sensitivity interleukin-6.

We further summarized the selected metabolites into a single composite metabolite score. In all subjects, the composite metabolite score (the first principal component) accounted for 42% of the variance in the contributing metabolites. S2 Fig shows loading of the composite metabolite score for each of the 6 metabolites. The composite score increased with higher HDL3 apolipoproteins, tyrosine, fatty acid with αCH2 and creatinine, and decreased with higher glucose and valine. In multivariable adjusted analysis, a 1-SD increase in the composite score was associated with a decrease in mtDNA content by 0.073±0.022 (*P* = 0.001).

In addition we found that mtDNA content was inversely associated with inflammatory markers including hs-CRP, hs-IL6, total white blood cell and neutrophil counts as well as neutrophil-to-lymphocyte ratio (Table 4).

## Discussion

The main finding of the present study was that in a general population lower peripheral blood mtDNA content was associated with higher levels of circulating metabolites indicative of disturbed lipid metabolism. Moreover, participants with higher levels of “classical” inflammatory markers such as hs-CRP, hs-IL-6 and neutrophil-to-lymphocyte ratio, had lower blood mtDNA content.

To our knowledge, no previous population study reported the association of circulating metabolites with the mtDNA content. Metabolites are a direct product of complex cellular biochemical processes. Novel high-throughput metabolomics approaches enable a simultaneous identification and quantification of circulating metabolites that can be related to different pathological conditions [19]. Several studies in humans demonstrated the usefulness of metabolite profiling for identifying the presence of cardiovascular diseases and predicting cardiovascular outcome [11, 12, 20, 21]. In 2,023 patients undergoing cardiac catheterization a higher baseline metabolomics score composed of fatty acid metabolites independently predicted total mortality and myocardial infarction [21]. Using 1H-NMR, Brindle *et al* measured serum metabolite spectra in 36 patients with severe coronary heart disease (triple vessel disease patients) and in 30 patients with angiographically normal coronary arteries [20]. In this study, metabolite derived composite scores distinguished coronary heart disease patients from controls with >90% sensitivity and specificity. Of notice, patients compared to controls had higher serum CH2 fatty acid side chains originating from triglyceride rich lipoproteins. In line with this finding, we found in our study a positive correlation between serum triglyceride levels and circulating fatty acid αCH2 chains (r = 0.64, *P* < 0.0001). This might imply a triglyceride origin of fatty acid metabolites that associated with a decline in peripheral blood mtDNA content in the present study.

In our study, decreased mtDNA content was also associated with higher serum lipid-free HDL3 apolipoproteins. Serum HDL is a versatile group of lipoproteins. Smaller, dense HDL3 particles have cholesterol clearing, anti-oxidative and anti-inflammatory properties that depend on the integrity on their surface apolipoproteins [22]. On the other hand, oxidative damage and chronic inflammation might deprive HDL of its cardiovascular protective effects [22, 23]. Indeed, apolipoproteins can be dissociated from the HDL particles and consequently accumulate in serum [24]. Recently, Shao *et al* demonstrated that myeloperoxidase, an enzyme produced by phagocytic inflammatory cells, oxidizes human apolipoprotein A-I [23]. Taken together, increase in lipid-poor HDL3 apolipoproteins that might indicate disturbed lipid metabolism in our population sample associated with lower peripheral blood mtDNA content. In our study, we also showed a strong correlation of tyrosine metabolite with HDL3 apolipoproteins (r = 0.95). Therefore, the observed association between mtDNA and tyrosine might be due to overlapping resonances arising from HDL3 apolipoproteins and tyrosine metabolites.

Compared to its nuclear counterpart mtDNA accumulates damage more extensively when exposed to oxidative stress [3, 25]. Both excessive ROS production as well as perturbed mitochondrial antioxidant capacity can disturb mtDNA replication and contribute to mtDNA content decline under pathologic conditions. Ide *et al* reported that myocardial ischemia in mice leads to excessive mitochondrial ROS production and decreased myocardial mtDNA content as compared to non-ischemic controls [4]. In this study, parallel decreases in mtDNA encoded transcripts and respiratory chain enzyme activities were observed in myocardial ischemia with no significant changes in levels and activity of nuclear encoded mitochondrial proteins. In addition, compared to healthy controls a 60% decrease in mtDNA amplification was detected in aortic tissue of apolipoprotein E deficient mice prior to developing detectable atherosclerotic lesions [3]. The disease process in atherosclerosis prone mice accentuated when mitochondrial antioxidant capacity was weakened by lower superoxide dismutase 2 activity [3]. Mitochondrial dysfunction accompanied by increased ROS production in rodents was also observed with renal failure and resulting creatinine accumulation [26]. In line with these experimental findings, higher peripheral blood mtDNA content was associated with lower risk of incident chronic kidney disease in 9058 participants from the Atherosclerosis Risk in Communities Study (hazard ratio 0.65; *P*<0.001) [27]. On the other hand, branched chain amino acid (BCAA) dietary supplementation including valine in mice upregulated expression mitochondrial ROS defence enzymes and promoted longevity [28]. In BCAA-supplement fed mice higher activity of inducers of mitochondrial biogenesis including the peroxisome proliferator-activated receptor γ coactivator-1α was observed with a concurrent increase in mtDNA content [28]. We found in our population sample a positive correlation between circulating valine and β-glucose levels (*P*<0.0001). Glucose can both fuel the ATP synthesis for valine stimulated assembly of new mtDNA as well as provide building blocks for nucleotide synthesis [29]. In line with these observations, in our participants circulating valine and glucose associated with higher peripheral blood mtDNA content.

Chronic low grade inflammation is reflected by increased levels of inflammatory markers including hs-IL6, hs-CRP and neutrophil-to-lymphocyte ratio. Recent experimental studies imply that mitochondrial dysregulation is intertwined with inflammatory processes [8, 30, 31]. Increased ROS production in mice during inflammation resulted in mtDNA damage, dysfunction and decreased mtDNA content [30]. Inflammation causes mitochondrial dysfunction that in turn can further stimulate the inflammatory process. Namely, increased mitochondrial ROS triggers the assembly of inflammasomes, multi-protein complexes that activate the inflammatory mediator cascade [8, 31]. In humans mitochondrial ROS activate inflammatory cells and induce production of IL-6 that is the main stimulator of protein synthesis, including CRP during the acute phase of inflammation [32, 33]. Of notice, IL-6 also mediates the transition from acute to chronic inflammation and therefore promotes chronic inflammation [33]. We previously identified the association between higher white blood cell counts and lower peripheral blood mtDNA content [14]. In line with our finding, a cross-sectional study in 94 healthy young adults reported a negative correlation between mtDNA content and hs-CRP levels [34]. In the present study, we also found that lower blood mtDNA content was also associated with higher IL-6 and neutrophil-to-lymphocyte ratio. Taken together, we confirm on a population level the interplay between mtDNA content and inflammation biomarkers but the precise mechanisms underlying these associations remain to be further unveiled.

Our study needs to be interpreted within the context of its limitations and strengths. First, all participants were white Europeans. Thus, the associations cannot be generalized to other ethnic or racial groups. Second, we measured the mtDNA content in peripheral blood buffy coat and its composition might vary with regard to counts of platelets and white blood cell. Nonetheless, blood samples were processed following the same protocol, and mtDNA content was standardized to the amount of nuclear DNA to minimize sample-to-sample variation. Third, that fragments in the nuclear genome known as nuclear mitochondrial insertion sequences (NUMTs) might affect accurate quantification of mtDNA content [35]. Therefore, ideally primer sets used for mtDNA quantification should be selected careful in order do not amplify such regions in the nuclear genome whether they are NUMTs or other repeat regions. In our study, we put special emphasis on specificity of the primers we used which would not bind to such repeat nuclear regions. Forth, as we used relative quantification to calculate the relative mtDNA content, the values do not represent the mtDNA copy number in the study population. Finally, the cross-sectional design of our study cannot determine a causal relationship between mtDNA content and levels of circulating metabolites and systemic inflammation.

## Conclusions

The present cross-sectional study demonstrated that peripheral blood mtDNA content in a general population is associated with a profile of circulating metabolites indicative of perturbed lipid metabolism, oxidative stress and inflammation. Further studies are necessary to clarify the molecular mechanisms governing this association and confirm its potential clinical usefulness.

## Supporting information

S1 Appendix Methods: Metabolite analysis and measurement of mtDNA content. (DOCX)

S1 Table Primer sequences and efficiencies for selected mitochondrial and nuclear amplification targets. (DOCX)

S2 Table Loading of latent factors. (DOCX)

S3 Table Associations between the mitochondrial DNA content and tyrosine regions. (DOCX)

S1 Fig Correlations between pairs of plasma metabolites. Panel A and panel B show pairwise correlation coefficients and significance of the correlations, respectively.
(DOCX)

S2 Fig Loading of the composite metabolite score. For tyrosine, HDL3 apolipoprotein, fatty acid with αCH2 and creatinine loading was positive (empty marker). For creatinine and β-glucose loading was negative (filled marker).
(DOCX)
