# Supplementary material for: Peripheral blood mitochondrial DNA content in relation to circulating metabolites and inflammatory markers: A population study
Source: PLoS One. 2017 Jul 13;12(7):e0181036. doi: 10.1371/journal.pone.0181036 (PMC5509283; doi:10.1371/journal.pone.0181036)
Supplement: S1 Appendix — (DOCX) [file pone.0181036.s001.docx]

**S1 Appendix**

**Methods**

**Metabolite analysis**

A 500 µl volume of each individual’s serum sample was mixed with 50 µl of a non-interfering solvent (deuterium oxide). A total of 500 µl of the mixture was individually transferred into a 5 mm high resolution NMR tube. The total preparation time for each sample was < 15 min. The 1H-NMR spectra were recorded with a Bruker Avance DRX 600 spectrometer (Rheinstetten, Germany) using a standard one-dimensional pulse sequence with water suppression. Samples were measured at 37°C. A total of 256 free induction decay (FID) signals were collected into 64 k data points with a spectral width of 14 ppm and a recycle delay of 1 s. Water signal was saturated with a weak irradiation during the recycle delay. Before Fourier transformation, the FID was multiplied by a 0.3 Hz exponential line broadening. Spectral chemical shift referencing on the Alanine CH3 doublet signal at 1.475 ppm was performed in all spectra. Resonances in these spectral regions were assigned using the literature and selected two-dimensional (2D NMR) spectra. We normalized all individual spectra to total spectral area to eliminate differences in total metabolite concentrations. We used available spectral databases and 2D NMR experiments to aid structural identification of relevant metabolites. All spectra were processed using Topspin 1.3 (Bruker Biospin GmbH, Germany) and transferred to MATLAB® (MathWorks Inc, 2006) using in-house scripts for data analysis. Signals belonging to selected metabolites were integrated and quantified using semi-automated in-house MATLAB peak-fitting routines based on Levenburg-Marquard optimization procedures. The target function for the optimization included experimental spectra measured for standard solutions of selected metabolites with complex multiple patterns and theoretically generated Lorentzian-shape signals for those metabolites with simpler spectral patterns.

**Measurement of mtDNA content**

*Real time quantitative PCR assay*

Genomic DNA was extracted from peripheral blood buffy coat, using the QIAmp DNA Mini Kit (QIAgen, Hilden, Germany), following the manufacturer’s instructions. Concentration and purity of the extracted DNA was determined using Nanodrop spectrophotometer (ND-1000, Isogen Life Science, De Meern, the Netherlands). The DNA samples were diluted to 2.4 ng/µL. We used a real time quantitative polymerase chain reaction (qPCR) to amplify two stable mitochondrial sequences and one housekeeping nuclear gene.^1,2^ For mitochondrial sequences, the forward and reverse primers were 5’-ATGGCCAACCTCCTACTCCT-3’ and 3’-CTACAACGTTGGGGCCTTT-5’ for MT-ND1, and 5’- CACCCAAGAACAGGGTTTGT-3’ and 3’- TGGCCATGGGTATGTTGTTAA-5’ for MTF3212/R3319. For the nuclear RPLP0 reference gene, the forward and reverse primers were 5’- GGAATGTGGGCTTTGTGTTC-3’ and 3’- CCCAATTGTCCCCTTACCTT-5’. The master mix contained Fast SYBR® Green dye 2x (Applied Biosystems, Foster City, CA, USA), forward and reverse primers diluted to 300nM per well, and RNAse–free water. Samples were run in triplicates on MicroAmp® Optical 384-well reaction plates. A single well contained 2.5 µL of the diluted DNA sample and 7.5 µL of the master mix. Each plate had 6 inter-run calibrators and 2 no-template controls to test for contamination. We amplified the target sequences in a 7900HT Fast Real-Time PCR thermal cycler (Applied Biosystems, Foster City, CA, USA). The thermal cycling profile was 20 s at 95°C for activation of the polymerase, followed by 40 cycles of 1 s at 95°C for denaturation and 20 s at 60°C for annealing and extension. A melting curve analysis was performed after each run to confirm the absence of non-specific products.

*Calculation of mtDNA Content*

The mtDNA content was calculated using qBase software (Biogazelle, Zwijnaarde, BE).^3^ First, we calculated mean threshold values (Ct) of individual triplicates and mean Ct values of each of the genetic sequence of all samples in all plates. The delta Ct was the difference between mean single triplet Ct value and mean Ct value averaged over all plates for each sequence. Relative quantities (RQ) with 100% primer efficiency equal 2delta Ct. Second, normalized relative quantities (NRQ) for each subject were calculated by dividing the corresponding RQ of each mitochondrial sequence with the RQ of the nuclear sequence. Finally, inter-run calibration was performed on a gene to gene basis. Calibration factors are based on geometric means of NRQ of inter-run calibrators. To calculate calibrated normalized relative quantities (CNRQ), sample NRQs on one plate were multiplied with the calibration factors. The final mtDNA content is the average CNRQ value of both mitochondrial sequences.

**References**

1. Janssen B, Munters E, Pieters N et al. Placental mitochondrial DNA content and particulate air pollution during in utero life. Environ Health Perspect 2012;120(9):1346-1352.

2. Knez J, Winckelmans E, Plusquin M et al. Correlates of peripheral blood mitochondrial DNA content in a general population. Am J Epidemiol 2015; in press.

3. Hellemans J, Mortier G, De Paepe A, Speleman F, Vandesompele J. qBase relative quantification framework and software for management and automated analysis of real-time quantitative PCR data. Genome Biol 2007;8:R19
