# Supplementary material for: Peripheral blood mitochondrial DNA content in relation to circulating metabolites and inflammatory markers: A population study
Source: PLoS One. 2017 Jul 13;12(7):e0181036. doi: 10.1371/journal.pone.0181036 (PMC5509283; doi:10.1371/journal.pone.0181036)
Supplement: S1 Table — (DOCX) [file pone.0181036.s002.docx]

**S1 Table.** Primer sequences and efficiencies for selected mitochondrial and nuclear amplification targets

| Sequence/gene | Location | Accession number | Forward 5’-3’ | Reverse 3’-5’ | Efficiency (%) |
| --- | --- | --- | --- | --- | --- |
| *MT-ND1* | M | NC_012920.1 | ATGGCCAACCTCCTACTCCT | CTACAACGTTGGGGCCTTT | 99.9 |
| *MTF3212/R3319* | M | NC_012920.1 | CACCCAAGAACAGGGTTTGT | TGGCCATGGGTATGTTGTTAA | 99.7 |
| *RPLP0* | N | NM_001002.3 | GGAATGTGGGCTTTGTGTTC | CCCAATTGTCCCCTTACCTT | 100 |

*MT-ND1,* mitochondrial encoded NADH dehydrogenase 1; *MTF3212/R3319* mitochondrial forward primer from nucleotide 3212 and reverse primer from nucleotide 3319; *RPLP0,* Ribosomal protein, large, P0; M, mitochondrial; N, nuclear. Accession numbers are from National Centre for Biotechnology Information ([www.ncbi.nlm.nih.gov](http://www.ncbi.nlm.nih.gov)).
