# Supplementary material for: Peripheral blood mitochondrial DNA content in relation to circulating metabolites and inflammatory markers: A population study
Source: PLoS One. 2017 Jul 13;12(7):e0181036. doi: 10.1371/journal.pone.0181036 (PMC5509283; doi:10.1371/journal.pone.0181036)
Supplement: S2 Table — (DOCX) [file pone.0181036.s003.docx]

| **S2 Table.** Loading of latent factors. | | | |
| --- | --- | --- | --- |
| **Latent factor** | **Factor 1** | **Factor 2** | **Factor 3** |
| **Amino Acids** |  |  |  |
| 2-Aminobutyrate | 0.006 | 0.194 | -0.078 |
| 4-Aminobutyrate | -0.111 | 0.117 | -0.198 |
| Alanine | 0.142 | 0.213 | -0.006 |
| Aspartate | -0.106 | 0.234 | 0.148 |
| Glutamate | -0.108 | 0.237 | 0.024 |
| Glycine | 0.083 | 0.129 | -0.131 |
| Glutamine | -0.196 | 0.113 | -0.157 |
| Isoleucine | 0.231 | -0.085 | -0.162 |
| Isoleucine and fatty acid with (-CH2)n | 0.036 | -0.186 | 0.030 |
| Leucine | -0.054 | 0.134 | -0.180 |
| Phenylalanine | -0.099 | 0.098 | -0.111 |
| Threonine | -0.054 | 0.116 | -0.113 |
| Tyrosine | -0.252 | -0.064 | -0.271 |
| Valine | 0.280 | -0.039 | -0.197 |
| **Lipids** |  |  |  |
| Fatty acid with α-CH2 | -0.298 | -0.054 | 0.216 |
| Fatty acid with =CH-CH2-CH2= + citrate+aspartate | 0.029 | 0.238 | 0.176 |
| Fatty acid with –CH3 | 0.270 | -0.083 | 0.030 |
| Fatty acid with =CH-CH2-CH2- | 0.034 | -0.030 | 0.245 |
| Fatty acid with –CH=CH | 0.034 | 0.018 | 0.248 |
| HDL3 apolipoproteins | -0.299 | -0.069 | -0.295 |
| Cholesterol | 0.191 | -0.135 | -0.155 |
| Valerate | -0.132 | -0.169 | 0.157 |
| Valerate + fatty acid with β-CH2 | -0.130 | 0.030 | 0.203 |
| **Carbohydrates** |  |  |  |
| α-Glucose | 0.228 | 0.085 | 0.011 |
| β-Glucose | 0.276 | 0.177 | -0.014 |
| Glucose | 0.098 | 0.208 | -0.064 |
| Glycoprotein | 0.010 | 0.068 | 0.177 |
| **Organic Acids** |  |  |  |
| Acetate | 0.026 | 0.189 | 0.005 |
| 3-Hydroxybutyrate | 0.077 | -0.177 | -0.068 |
| 4-Hydroxybutyrate | -0.060 | 0.164 | -0.100 |
| Lactate | -0.023 | -0.028 | 0.236 |
| 2-Oxobutyrate | 0.034 | 0.172 | 0.255 |

| **Other Metabolites** | | | |
| --- | --- | --- | --- |
| Creatinine | -0.341 | 0.098 | 0.068 |
| Choline + phosphocholine | -0.021 | 0.058 | 0.003 |
| Ethanolamine | -0.073 | 0.248 | 0.057 |
| Trimethylamine | -0.026 | 0.082 | -0.155 |
| Glycerol | -0.068 | 0.159 | -0.078 |
| Ethanol | -0.007 | 0.074 | -0.125 |
| Creatine + creatine-phosphate | -0.148 | 0.132 | -0.183 |
| Phosphocholine | 0.037 | -0.041 | -0.189 |
| Glucose + 2-aminobutyrate | 0.213 | 0.192 | -0.082 |
| Glucose + glutamine | 0.119 | 0.321 | 0.104 |
| Glucose + 2-Phosphoglicerate | -0.020 | 0.164 | -0.073 |
| Unknown molecule | 0.041 | 0.212 | -0.017 |
| Factors 1, 2 and 3 were derived by partial least square analysis of the 44 metabolites. | | | |
