# Supplementary material for: Peripheral blood mitochondrial DNA content in relation to circulating metabolites and inflammatory markers: A population study
Source: PLoS One. 2017 Jul 13;12(7):e0181036. doi: 10.1371/journal.pone.0181036 (PMC5509283; doi:10.1371/journal.pone.0181036)
Supplement: S3 Table — (DOCX) [file pone.0181036.s004.docx]

| **S3 Table.** Associations between the mitochondrial DNA content and tyrosine regions | | | | | | | | | | |  |  |
| --- | --- | --- | --- | --- | --- | --- | --- | --- | --- | --- | --- | --- |
| **Explanatory variable** | | Residual water peak region (ppm) | | **mtDNA content** | | | | | | | | |
|  |  |  |  | *Parameter estimate ± SE | | 95% CI | | *P* value | | *P** value | | |
| Tyrosine region 1 | 3.05 ̶ 3.07 | | -0.17±0.16 | | -0.49 to 0.14 | | 0.28 | | 0.18 | | |  |
| Tyrosine region 2 | 3.17 ̶ 3.18 | | -0.26±0.21 | | -0.68 to 0.17 | | 0.23 | | 0.18 | | |  |
| Tyrosine region 3 | 3.93 ̶ 3.95 | | -0.16±0.20 | | -0.55 to 0.24 | | 0.44 | | 0.41 | | |  |
| Tyrosine region 4 | 6.88 ̶ 6.90 | | -0.33±0.14 | | -0.61 to -0.063 | | 0.016 | | 0.021 | | |  |
| Tyrosine region 5 | 7.17 ̶ 7.21 | | -0.34±0.14 | | -0.62 to -0.068 | | 0.015 | | 0.020 | | |  |
| Explanatory variables were normalized by a logarithmic transformation. Parameter estimates, corresponding SE and 95%CI are associated with a doubling of the metabolites. *P* values are for models with explanatory variables normalized by a logarithmic transformation. *P** values are for models with explanatory variables normalized by a rank transformation. mtDNA, mitochondrial deoxyribonucleic acid; SE, standard error; CI, confidence interval. | | | | | | | | | | |  |  |
