# Supplementary material for: Peripheral blood mitochondrial DNA content in relation to circulating metabolites and inflammatory markers: A population study
Source: PLoS One. 2017 Jul 13;12(7):e0181036. doi: 10.1371/journal.pone.0181036 (PMC5509283; doi:10.1371/journal.pone.0181036)
Supplement: S1 Fig — Panel A and panel B show pairwise correlation coefficients and significance of the correlations, respectively. (DOCX) [file pone.0181036.s005.docx]

| **S1 Figure.** Correlations between pairs of plasma metabolites. Panel A and panel B show pairwise correlation coefficients and significance of the correlations, respectively. | |
| --- | --- |
| A) | B) |
|  |  |
