# Supplementary material for: Peripheral blood mitochondrial DNA content in relation to circulating metabolites and inflammatory markers: A population study
Source: PLoS One. 2017 Jul 13;12(7):e0181036. doi: 10.1371/journal.pone.0181036 (PMC5509283; doi:10.1371/journal.pone.0181036)
Supplement: S2 Fig — For tyrosine, HDL3 apolipoprotein, fatty acid with αCH2 and creatinine loading was positive (empty marker). For creatinine and β-glucose loading was negative (filled marker). (DOCX) [file pone.0181036.s006.docx]

| **S2 Figure.** Loading of the composite metabolite score. For tyrosine, HDL3 apolipoprotein, fatty acid with αCH2 and creatinine loading was positive (empty marker). For creatinine and β-glucose loading was negative (filled marker). |
| --- |
|  |
|  |
